# *Lankesterella* and *Isospora* Coccidians: Differences in Host Specificity of Blood Parasites in Passerines

**DOI:** 10.3390/microorganisms13040743

**Published:** 2025-03-26

**Authors:** Ashwin Kumar Saravana Bhavan Venkatachalam, Jana Brzoňová, Milena Svobodová

**Affiliations:** Department of Parasitology, Faculty of Science, Charles University, 128 43 Prague, Czech Republic; jana.brzonova@natur.cuni.cz

**Keywords:** avian blood parasites, coccidia, Eimeriidae, passerines, host–parasite interactions, host specificity, *Atoxoplasma*

## Abstract

Parasitic protozoa such as *Lankesterella* and *Isospora* are common in avian hosts, particularly in passerines. Despite their high prevalence, the diversity of these parasites within avian populations remains poorly understood. This study aimed to assess the diversity of *Lankesterella* and *Isospora* in passerine birds, using the SSU rRNA gene to characterize and compare the genetic variation in both parasites across multiple avian host species. For *Isospora*, the extraintestinal blood stages and oocysts from feces were compared. Minimum spanning networks were constructed to visualize haplogroups in relation to host specificity and to reveal the identity of various developmental stages. A total of 122 sequences from eight passerine species were used to generate a haplotype network for *Lankesterella*, and a total of 103 sequences (64 from blood and 39 from feces) was used for *Isospora*. We detected a total of 36 haplotypes for *Lankesterella* and 33 haplotypes for *Isospora*. In *Lankesterella*, we confirmed that the sedge warbler has its own specific lineages, whereas other warbler species share lineages belonging to three haplogroups; blue, great, marsh and willow tits have their own unique groups of lineages. *Isospora* is less host-specific than *Lankesterella* in avian hosts; nevertheless, *Isospora* sequences from blood and feces were identical in their respective hosts. Our findings provide insights into the diversity and host specificity of blood coccidians; moreover, we molecularly characterized the developmental stages of *Isospora*.

## 1. Introduction

Avian blood protists frequently occur in a variety of passerine host species. Out of those belonging to Apicomplexa, haemosporidian parasites such as *Plasmodium*, *Haemoproteus*, and *Leucocytozoon* have been commonly studied, while others such as *Lankesterella* and *Isospora* have been neglected. The life cycles of these parasites are insufficiently known, partly due to the fact that their developmental stages look similar in the hosts [1]. Despite previous research on avian *Lankesterella* and *Isospora* [2,3,4,5,6], the genetic diversity and host–parasite relationships of these blood parasites in avian populations remain understudied.

The genus *Lankesterella*, which belongs to the family Lankesterellidae [7], was first described from amphibians [8] and later reptiles [9]. Recent studies have found out that avian infections are not an exception [2,3,10,11]. In older studies, avian *Lankesterella* was mistakenly reported as *Hepatozoon* [12] but was shown to be related to amphibian lankesterellids [4]. *Lankesterella* are considered to be host-specific [2,11,13] and have a heteroxenous life cycle, with mites and mosquitoes being putative vectors [3]. The role of these vectors, however, remains poorly understood. This is particularly important for understanding the epidemiology of *Lankesterella* infections in wild bird populations.

The genus *Isospora* belongs to the family Eimeriidae [7]. Passerine coccidia that are excreted in the feces are predominantly placed within the genus *Isospora*, but there are also reports of the occurrence of other coccidians in passerines belonging to the genera *Eimeria* and *Caryospora* [14]. *Isospora* has had a taxonomically confusing history [15] since their blood stages were previously assigned to the genus *Atoxoplasma*, which is now mostly considered a synonym of *Isospora* [16,17]. *Isospora* parasites are usually detected as fecal oocysts (intestinal stage) or as merozoites in the blood of avian host [18]. Transmission occurs via the ingestion of sporulated oocysts from the host feces [16,19]. *Isospora* spp. are considered host-specific, as revealed both by phylogenetic studies and experimental transmissions, but some potential for spillover or host-switching still exists [2,15,18,20,21,22]. The extent to which these spillover events contribute to parasite diversification remains debatable.

The host species selected for our study included eight passerine species: four cavity-nesting species that are resident/short-distance migrants belonging to the Paridae (great tit (*Parus major*), blue tit (*Cyanistes caeruleus*), marsh tit (*Poecile palustris*) and willow tit (*P. montanus*)), and four open-nesting species that are long-distance migrants from the family Acrocephalidae (sedge warbler (*Acrocephalus schoenobaenus*), reed warbler (*A. scirpaceus*), marsh warbler (*A. palustris*), and great reed warbler (*A. arundinaceus*)) [23]. These passerine species occur sympatrically and are known hosts of *Lankesterella*, while in warblers, *Isospora* blood stages were found as well, contrary to tits, where blood stages have not been detected so far. Apart from these model species, we included other passerine hosts with records of *Isospora* in blood, namely hawfinch (*Coccothraustes coccothraustes*) and chaffinch (*Fringilla coelebs*). By including a broad range of host species, our study aimed to assess whether the phylogenetic relatedness between hosts influences parasite lineage sharing, thereby providing a deeper understanding of parasite–host relationships. In addition to confirming the identity of the parasite stages of the *Isospora* found in the blood and feces of the respective hosts, we were interested in the patterns of host specificity in these two parasite genera.

The aims of our study were 1. to compare the host specificity of the two closely related coccidian genera and 2. to find out if the *Isospora* blood and fecal stages correspond molecularly. We supposed that (i) *Lankesterella* are host-specific at the species or genus level and (ii) *Isospora* host specificity is related to the differences in their developmental type (strictly intestinal versus extraintestinal lineages). By investigating these patterns, we hope to shed light on how parasite host specificity affects host–parasite interactions. We studied the two parasite genera in the same set of host populations, thus avoiding the bias potentially generated by factors such as habitat differences, environmental conditions, or transmission patterns.

## 2. Methodology

### 2.1. Field Work and Sampling

Birds were trapped and ringed during the breeding season (April–July) from 2014 to 2022 using mist nets or in nest boxes, as previously described [11,24] in several localities in Czechia. The bird species, age, and sex were determined by experienced ringers. Blood was taken from the metatarsus vein articulation (*vena metatarsalis plantaris superficialis media*); 10–20 μL of blood was stored in 96% ethanol until further use. Blood sampling was carried out under permits 50982/ENV/14-2961/630/14 and MZP/2019/630/1081 of the Ministry of the Environment. For fecal sample collection, captured birds were kept in clean textile bags for approximately 30 min and released. Feces were collected from 12 am onwards due to the circadian rhythms of oocyst shedding [14] and, if available, dissolved in a 2% aqueous solution of potassium dichromate (K_2_Cr_2_O_7_) and stored at room temperature. All bird captures and manipulations were carried out by licensed workers.

### 2.2. Parasite Detection Methods

In the model host species, PCR was used as the primary detection method. The rarer host species were included based on large-scale microscopical detection; Giemsa-stained blood smears were checked at 1000× for 10 min; samples positive for *Lankesterella/Isospora* stages microscopically were subject to PCR. DNA from bird blood was isolated; a nested PCR protocol targeting the SSU rRNA gene was used for *Lankesterella* and *Isospora* detection [11] with the following primers: EF (5′-GAAACTGCGAATGGCTCATT-3′) and ER (5′-CTTGCGCCTACTAGGCATTC-3′) for the first step [25], and Hep153F (50-GTAATTCTATGGCTAATACATGCGC-30) and Hep1496R (50-TTATTGCCTCAAACTTCCTTGCG-30) for the second step [11], and 1 mL of the initial PCR product. To avoid cross-contamination, the DNAs from a single host species were used in individual PCR runs. A negative control was used for every 10 samples to ensure the accuracy of the experimental results. Blood positive for *Lankesterella* was used as a positive control. PCR products were analyzed on 1% agarose gels, stained with SybrSafe, visualized under UV light, purified using ExoSAP-IT TM PCR Product Clean up Reagent (Thermo Scientific, Waltham, MA, USA), and sequenced with Hep153F primer using an Applied Biosystems^®^ 3500 Genetic Analyzer (Waltham, MA, USA) at the core facility of the Faculty of Science, Charles University. For PCR detection of *Isospora* from feces, the samples were first checked under a microscope at 200× magnification. Samples positive for *Isospora* oocysts (39/79) were washed three times in saline solution to wash out the potassium dichromate. For DNA isolation, Qiagen DNeasy PowerSoil kit (Hilden, Germany) was utilized according to the manufacturer’s protocol, and the PCR protocol describe above was used for detection.

### 2.3. Haplotype Network Analysis

Two SSU rRNA haplotype networks were created for both *Lankesterella* and *Isospora*. A minimum of three sequences (if available) of the parasite (*Lankesterella/Isospora*) from the model host species were selected for the construction of the haplotype networks. For *Lankesterella*, a total of 122 of our own sequences of model host populations of passerines originating from Czechia was used, which included 41 sequences from warblers and 81 sequences from tits. For *Isospora*, the sequences obtained from both the extraintestinal (64) and intestinal (39) stages of various passerine hosts were used to generate the haplotype network. The sequences were uploaded, aligned using MUSCLE, and edited in Geneious prime (V. 2024.0.7). The quality of the sequences was checked in Geneious Prime, and a few sequences from warbler samples (12/72) showed double peaks. To correct them, heterozygous sites were identified in the chromatogram and aligned with a clean reference sequence, after which the bases were manually edited. The alignment was then generated as a .nexus file. DnaSP, V. 6.02.13 (DNA sequence polymorphism) [26], was used to generate a haplotype file. A network of 815 bp SSU rRNA partial sequences was generated for both *Lankesterella* and *Isospora*. A minimum spanning haplotype network was generated with PopART [27] from the previously generated haplotype file to depict intraspecific variations based on the genetic differences. The networks were graphically prepared and provided with information on host species. The networks were then edited in Inkscape 1.1.2 (Inkscape Project 2024). An extended *Lankesterella* haplotype network of 815 bp SSU rRNA sequences was generated with all available sequences from GenBank.

### 2.4. Phylogenetic Analysis

To provide an overview of the diversity of the lineages, maximum likelihood (ML) trees were constructed for both the *Lankesterella* and *Isospora* sequences that were obtained from passerine hosts. A data set containing 107 18S rRNA gene sequences was used for the phylogenetic analysis, out of which 46 were newly obtained avian *Lankesterella* sequences (17 warbler and 27 tit sequences). Four to seven *Lankesterella* sequences (if available) per haplogroup were selected (Table S1); moreover, occasional findings from other hosts and all available avian *Lankesterella* sequences from GenBank were used in the analysis. An *Isospora* sequence from a passerine host was used as an outgroup. The phylogenetic tree was constructed as described in [11]. Statistical support was assessed by 1000 bootstrap pseudoreplicates in RAxML. For *Isospora*, the same methodology was used as previously described, with a dataset containing 78 SSU sequences, of which 36 were newly obtained *Isospora* sequences from the blood and fecal samples of passerine hosts. Two to four sequences (if available) per haplogroup per sample type (blood/feces) were selected (Table S2). A passerine *Lankesterella* sequence was used as an outgroup. All sequences were uploaded to NCBI GenBank and can be retrieved using accession numbers PQ765534–PQ765577, PQ765739–PQ765740 (*Lankesterella*) as well as PQ772231–PQ772261 and PV000754–PV000758 (*Isospora*).

## 3. Results

**Figure 1:**
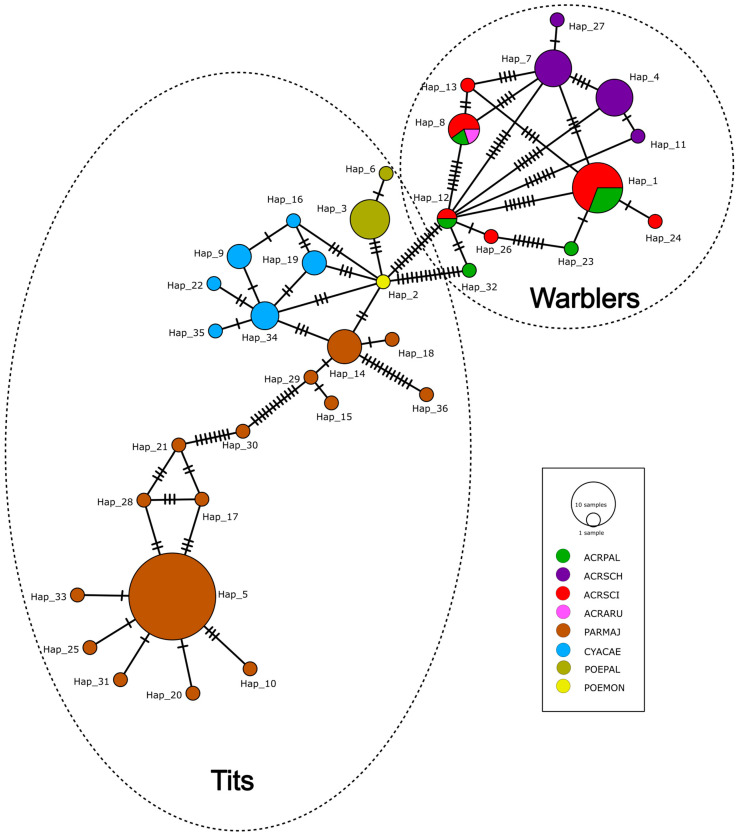
Minimum spanning DNA haplotype network of SSU rRNA sequences of avian *Lankesterella* lineages obtained from blood of passerine hosts. Each circle represents a unique haplotype/lineage. The size of the circle indicates the frequency of sequences in each haplotype group. Hatchets indicate the number of nucleotide base pair differences between each group. The color of the circles denotes the studied model species (shown in legend; abbreviations in legend shown in Table S5). Groups with similar haplotypes that belong to the same family are framed within dotted lines.

We found distinct patterns in the host specificity of passerine *Lankesterella* and *Isospora*. The *Lankesterella* haplotype network included 122 sequences that were obtained from eight model species belonging to two passerine families, namely, the sedge, reed, marsh and great reed warblers from the Acrocephalidae; the blue, great, marsh, and willow tits from the Paridae. No *Lankesterella* sequence was obtained from any host feces positive for oocysts. For host specificity, *Lankesterella* seem to be specific at the host species level with the exception of the genus *Acrocephalus*, where three species (the reed, marsh, and great reed warblers) share lineages that form three major haplogroups (Figure 1; Hap_1, Hap_8, Hap_12), while the sedge warbler hosts its own specific haplogroups (Figure 1; Hap_4, Hap_7). On the other hand, the host specificity of the *Lankesterella* marsh and willow tits seems to be at the host species level, since each of the two congeneric species has its own lineages (Figure 1; Hap_3,6 (marsh tit) and Hap_2 (willow tit)). This pattern is still evident in an extended haplotype network that includes ours as well as published sequences of avian *Lankesterella* from a broader range of hosts (Figure S1, Table S3).

**Figure 2:**
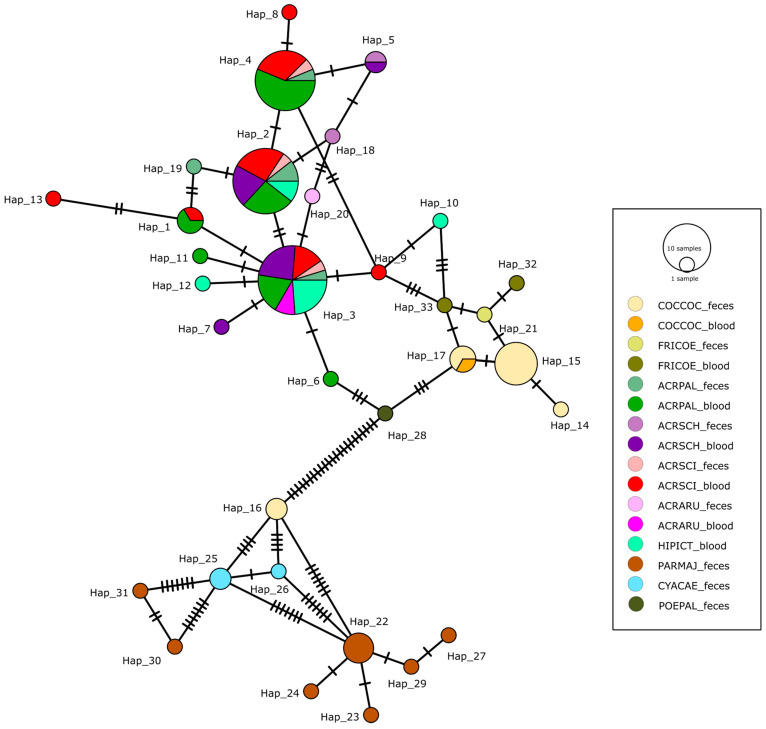
Minimum spanning DNA haplotype network of SSU rRNA sequences of avian *Isospora* lineages isolated from blood and fecal samples of passerine hosts. Each circle represents a unique haplotype/lineage. The size of the circles indicates the frequency of sequences in each haplotype group. Hatchets indicate the number of nucleotide base pair differences between each group. The color of the circles denotes the studied model species (shown in legend; abbreviations in legend shown in Table S5).

The *Isospora* sequences were obtained from both blood and fecal samples. The sequences of *Isospora* from blood comprised a total of 64 sequences obtained from seven passerine species: reed, sedge, marsh, great reed, icterine warblers, hawfinch, and chaffinch. A total of 39 sequences were obtained from the fecal samples from nine passerine species: the reed, sedge, marsh, great reed warblers, great, blue, willow tits, hawfinch, and chaffinch. No *Isospora* sequences were detected in the blood of tits. The *Isospora* sequences obtained from the blood and feces of different species of warblers represent three main haplogroups where the lineages are shared between the species, including not only the sedge warbler but also the icterine warbler (*Hippolais icterina*) (Figure 2). The sequences from the feces and blood were identical in the respective haplogroups. Apart from the warbler sequences, the *Isospora* sequences obtained from the different genera of tits, the hawfinch (*Coccothraustes coccothraustes*), and chaffinch (*Fringilla coelebs*) had their own unique haplotypes (Figure 2). Interestingly, we also found that one haplotype from the hawfinch was closely related to the *Isospora* of Paridae, while a single haplotype from marsh tit feces did not cluster with the other haplotypes of the Paridae (Figure 2; Hap_28). The prevalences based on the microscopical records of *Lankesterella* and *Isospora* in infrequent passerine hosts in various localities in Czechia were assessed (Table S4). A total of 995 blood smears from eight infrequent species of passerines were microscopically screened for the presence of *Isospora*/*Lankesterella*, of which only one individual/species was positive for either parasite with a prevalence range of 0.84% for *Isospora* and 3.4% for *Lankesterella* (Table S4).

**Figure 3:**
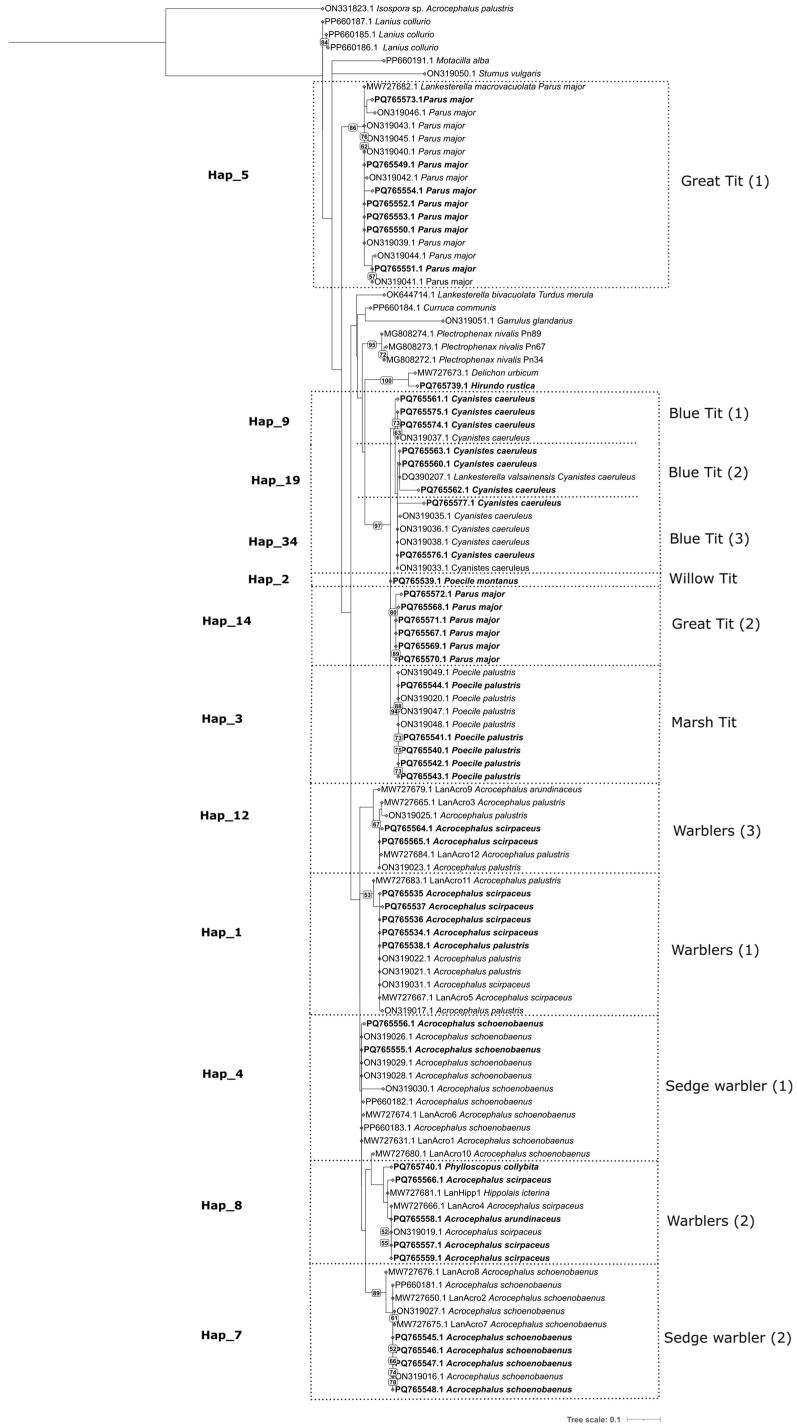
Phylogenetic tree of avian *Lankesterella* spp. based on SSU rRNA gene sequences. The tree was constructed using the maximum likelihood in RAxML 8.0.0 (GTRGAMMAI model). Bootstrap values are shown at the branches (only support values >50 are indicated). A sequence of *Isospora* sp. from a sedge warbler is included as an outgroup. Newly determined sequences are highlighted in bold. Respective haplotype numbers are shown on the right side of the tree.

**Figure 4:**
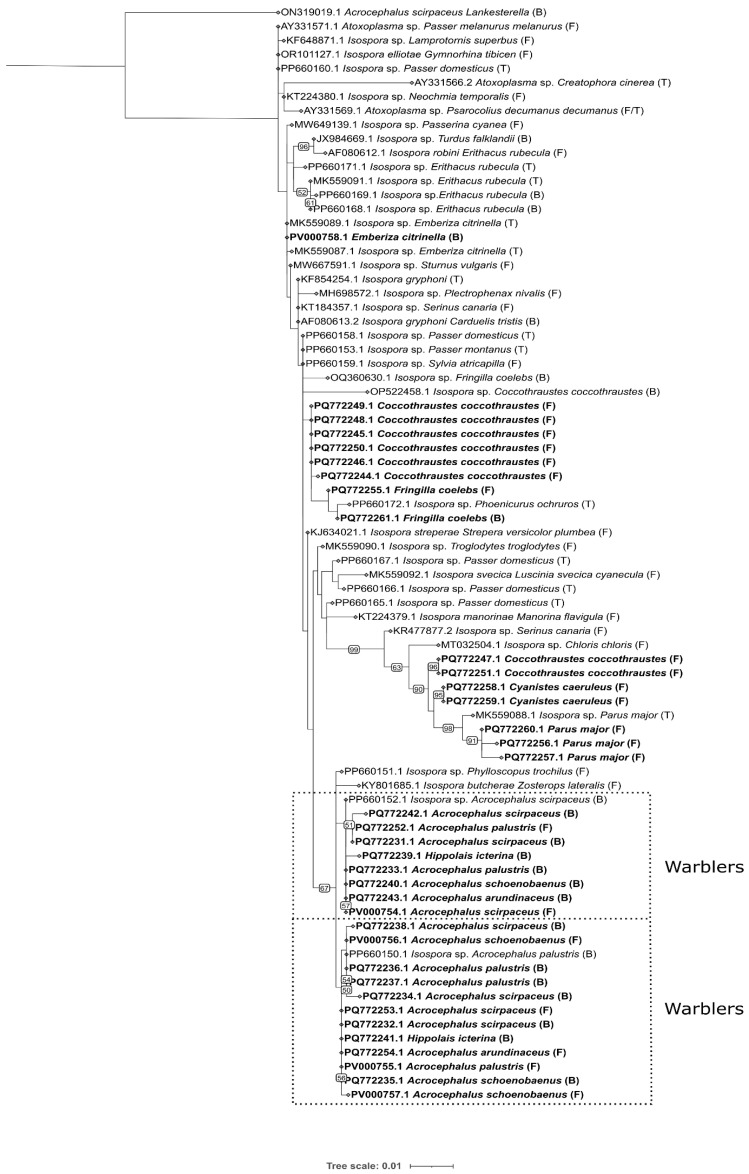
Phylogenetic tree of avian *Isospora* spp. based on SSU rRNA gene sequences (18S). The tree was constructed using the maximum likelihood in RAxML 8.0.0 (GTRGAMMAI model). Bootstrap values are shown at the branches (only support values > 50 are indicated). A sequence of *Lankesterella* sp. from sedge warbler is included as an outgroup. Newly obtained sequences are highlighted in bold. Sample type denoted as blood (B); feces (F); tissue (T).

Two phylogenetic trees were constructed with our newly obtained sequences of *Lankesterella* and *Isospora* and all available respective 18S rRNA sequences from GenBank (Figure 3 and Figure 4). The results were in concordance with the haplotype networks. For *Lankesterella*, the sequences obtained from the great tit formed two distinct clades and so did the sequences from the sedge warbler. The *Lankesterella* sequences from the reed, marsh, and great reed formed three different clades, each containing the sequences from all three respective hosts (Figure 3), which is in concordance with the haplotype network (Figure 1 and Figure S1). Our new sequence from the common chiffchaff (*Phylloscopus collybita*) clusters with generalist *Acrocephalus* lineages but represents a unique haplotype (see Figure 1 and Figure S1). For *Isospora*, a phylogenetic tree with all available sequences showed that *Acrocephalus* warblers had two different haplogroups irrespective of the sample type (blood/feces) (Figure 4). While most of the *Isospora* sequences from the hawfinch cluster with the chaffinch and other host sequences, two sequences from the hawfinch fall into a clade that includes great and blue tit sequences as well (Figure 4). The icterine warbler sequences clustered with the *Acrocephalus* warbler sequences within two major haplogroups (Figure 2; Hap_3, Hap_2).

## 4. Discussion

In this study, we focused on determining the host specificity of *Lankesterella* and *Isospora* in passerines and revealed that their host specificity differs between the studied model hosts at some points but has similar patterns in others. Previous studies of blood parasites in passerine hosts that have used haplotype networks have only dealt with haemosporidians [28,29]. Our results confirm, on a larger data scale (Tables S4 and S5), that *Lankesterella* has high host specificity towards the sedge warbler, whereas the other genera of warblers share their lineages [3,11]. On the other hand, the warbler *Isospora* are shared, including the sedge warbler, revealing a different host specificity pattern. In the haplogroups that contained a sufficient number of samples, the sequences from blood and oocyst stages were shared between the hosts of the family Acrocephalidae, confirming thus that they represent different stages of the parasite life cycle. The *Lankesterella* haplotype network that included an extended number of hosts showed similar patterns of host specificity, and these results were also in concordance with the phylogenetic tree. The presence of host-specific lineages in certain species suggests a long-term evolutionary association between the parasite and their avian hosts, potentially driven by various ecological and behavioral factors that could influence transmission dynamics [30]. For the extended haplotype network analysis of *Lankesterella* lineages, the alignment had to be trimmed since the original sequence lengths from GenBank were too long; as a result, some data were lost, and we had some minor differences in the haplotype networks (Figure S1 and Figure 1).

*Lankesterella* has strong host specificity at the genus level that sometimes extends to the host species level. Similar patterns of high host specificity were also observed in previous studies on *Lankesterella* phylogeny [3,11]; however, host switches were reported [2]. The number of lineages obtained from congeneric species (*Poecile*) in our study was unfortunately too low to make a definitive conclusion. Notably, the sedge warbler hosts unique lineages of *Lankesterella*, while other warbler species share lineages [3,11]. With additional warbler sequences, we were able to identify that the sedge warbler hosted two unique haplogroups (Figure 1; Hap_4, Hap_7). This pattern of related host species sharing lineages of the parasite could indicate close evolutionary relationships among the haplotypes associated with phylogenetically similar hosts, while distantly related hosts have distinct lineages [2,11].

*Isospora* lineages usually have high host specificity [2,15]. *Isospora* can sometimes exhibit moderate host specificity in cases where closely related bird species share similar ecological niches, allowing the parasite to occasionally infect multiple hosts within the same avian genus, thus indicating complex dynamics of host–parasite specificity [2,15,20]. It seems that, in some cases, the host (un)specificity extends even across passerine families.

The *Isospora* sequences were obtained from both blood and fecal samples (extraintestinal and intestinal stages), highlighting a life cycle that is different from *Lankesterella.* We found that *Isospora* lineages are shared between blood and feces in the respective hosts; these results are in concordance with a single host species study on blackcap (*Sylvia atricapilla*) [31] based on sequences from the mitochondrial COI gene. Interestingly, we did not obtain any *Isospora* sequences from the blood of tits, even though many individuals were screened by PCR (202 great tits, 187 blue tits, and 56 marsh tits; [10]). Different types of development (extraintestinal, strictly intestinal) have been described for two *Isospora* species infecting a single host, the canary [19]. While avian *Isospora* are monoxenous, many of them possess extraintestinal stages; however, it seems that the *Isospora* species from tits exhibit a strictly intestinal type of development. In this context, there are two groups of lineages originating from hawfinches that are placed on distant positions in the phylogenetic tree, one falling into the lineages with extraintestinal stages and the second one clustering with tit lineages, suggesting it might have no extraintestinal stages since it was not detected from blood smears. Thus, from our data, it seems that *Isospora* with extraintestinal and strictly intestinal stages might form different clades.

In some cases, having more lineages from certain host species might have been useful. However, the prevalences in some hosts are very low [2,3,32], or birds are trapped at an inappropriate time of the day when there is no oocyst shedding due to the circadian rhythms of coccidians [14,33,34]. Oocyst shedding is usually at its peak during the evening (16:00–18:00) due to the diurnal variation in *Isospora* oocysts’ shedding. The intensity of oocysts production is also higher in juveniles compared to adults as coccidia infection rapidly decreases with host age [34]. We were able to compare blood and fecal samples from most of our model hosts except for the icterine warbler and *Poecile* tits; while tits are rarely trapped in general, the icterine warbler was caught only in the morning due to constant effort site (CES) methodology but was found with very high *Isospora* oocyst prevalences in previous studies [32,34,35]. Even for some common passerine species, we obtained only single sequences regardless of the substantial number of individuals examined. It should be mentioned that prevalences can be influenced by the locality of sampling, which can be the most significant factor that influences prevalence [10], and by the feeding behavior/foraging ecology of bird species [34], which might explain the prevalence differences among studies. The microscopically assessed prevalences of blood stages in infrequent hosts are generally very low when compared to the prevalences in model hosts (Table S4) even if microscopy is less sensitive in *Lankesterella* detection than PCR, with 13% vs. 23% of warblers and 21% vs. 29% tits being positive using microscopy and PCR, respectively ([36], Svobodová, unpublished).

## 5. Conclusions

We found strong host specificity in *Lankesterella* and *Isospora* among model passerines at the host genus or even the host species level in the case of *Lankesterella*. Our findings support the hypothesis that *Lankesterella* exhibits host specificity at the species or the genus level, with the exception of the *Acrocephalus* warblers, where several species share lineages, while the sedge warbler has unique lineages. For *Isospora*, the sharing of haplotypes among the *Acrocephalus* warblers including the sedge warbler shows that *Isospora* are less host-specific than *Lankesterella*. Some *Isospora* groups are related to the differences in their developmental type: the haplotypes from hawfinch cluster in two different clades based on their supposed type of development; those with blood stages cluster with the chaffinch, while those without blood stages cluster with the tits. This supports our hypothesis that *Isospora* host specificity is related to the differences in their developmental type (strictly intestinal versus extraintestinal lineages). Future studies should explore the ecological and evolutionary drivers of these patterns, along with integrating both molecular- and microscopy-based approaches, with experimental validation, which will be crucial for refining our understanding of the host–parasite relationships shaping avian coccidian diversity.

## Data Availability

The sequence data presented in the study are openly available in GenBank (https://www.ncbi.nlm.nih.gov/genbank/, accessed on 19 March 2025), accession numbers: PQ765534–PQ765577, PQ765739–PQ765740 (*Lankesterella*) as well as PQ772231–PQ772261 and PV000754–PV000758 (*Isospora*).

## Supplementary Materials

The following supporting information can be downloaded at https://www.mdpi.com/article/10.3390/microorganisms13040743/s1: Figure S1: Minimum spanning DNA haplotype network of all available avian *Lankesterella* SSU rRNA sequences; Table S1: Summary of major *Lankesterella* haplotypes and their distribution across passerine hosts; Table S2: Summary of major *Isospora* haplotypes collected from feces/blood and their distribution across passerine hosts; Table S3: *Lankesterella* lineage comparison with haplotypes; Table S4: Prevalence of *Lankesterella* and *Isospora* in infrequent hosts; Table S5: Model hosts and sample type.
