# Supplementary material for: Lankesterella and Isospora Coccidians: Differences in Host Specificity of Blood Parasites in Passerines
Source: Microorganisms. 2025 Mar 26;13(4):743. doi: 10.3390/microorganisms13040743 (PMC12029294; doi:10.3390/microorganisms13040743)
Supplement: Supplementary file 1 [file microorganisms-13-00743-s001.zip › microorganisms-3500442-supplementary.pdf]

Supplementary

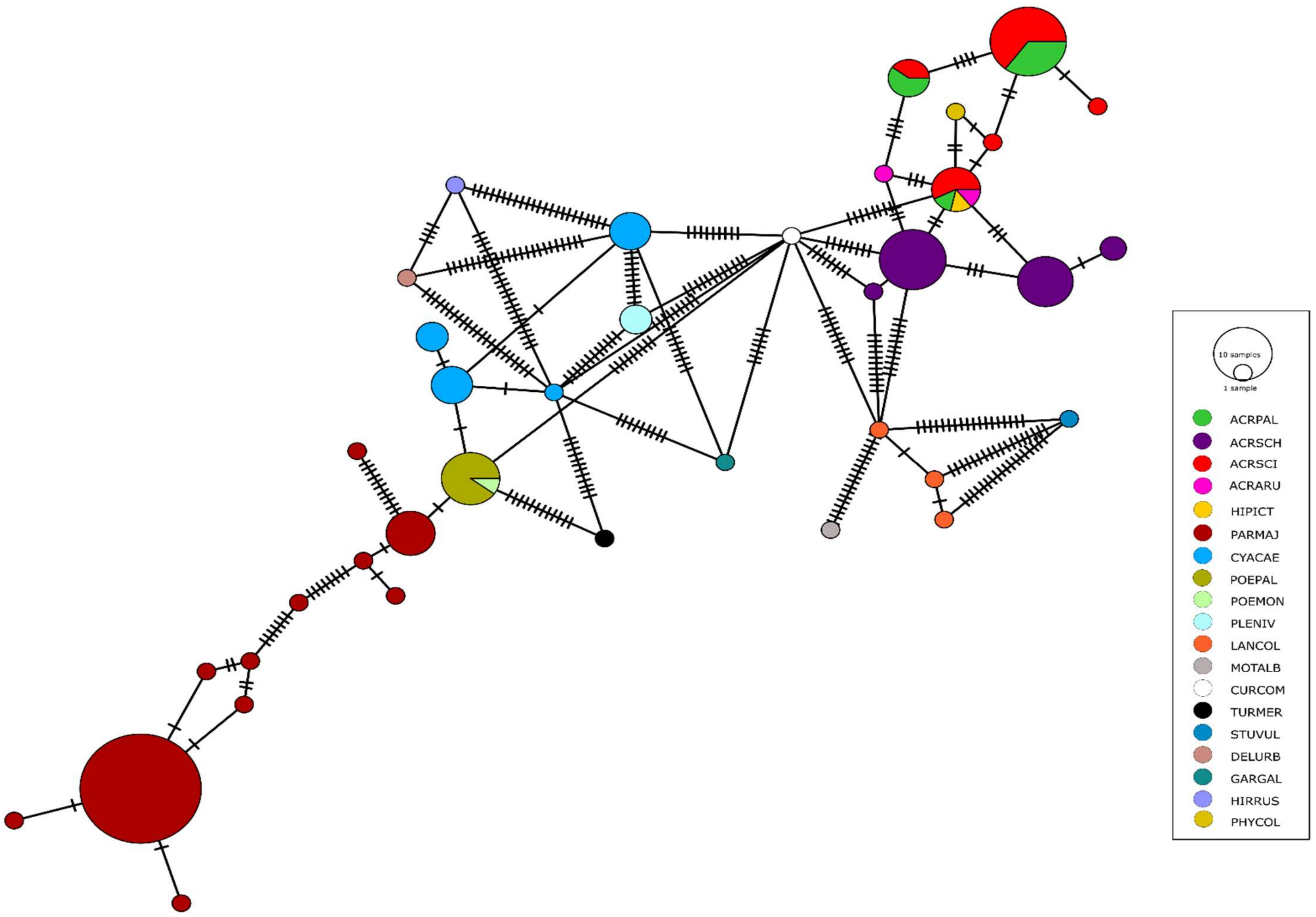

Figure S1. Minimum spanning DNA haplotype network of all available avian *Lankesterella* SSU rRNA sequences isolated from blood of passerine hosts. Each circle represents a unique haplotype/lineage. The size of the circles indicates the frequency of sequences in each haplotype group. Hatchets indicate the number of nucleotide base pair differences between each group. Colour of the circles denotes the studied model species (Shown in legend).

Table S1. Summary of major *Lankesterella* haplotypes and their distribution across passerine hosts. Haplotypes that occur more than once are included in the table.

Haplotype	ACRSCI	ACRPAL	ACRSCH	ACRARU	PARMAJ	CYACAE	POEPAL	POEMON	Total Sequences
Hap_1	9	4	-	-	-	-	-	-	13
Hap_3	-	-	-	-	-	-	8	-	8
Hap_4	-	-	7	-	-	-	-	-	7
Hap_5	-	-	-	-	39	-	-	-	39
Hap_7	-	-	7	-	-	-	-	-	7
Hap_8	3	1	-	1	-	-	-	-	5
Hap_9	-	-	-	-	-	3	-	-	3
Hap_12	1	1	-	-	-	-	-	-	2
Hap_14	-	-	-	-	6	-	-	-	6
Hap_19	-	-	-	-	-	3	-	-	3
Hap_34	-	-	-	-	-	4	-	-	4

Table S2. Summary of major *Isospora* haplotypes collected from feces/blood and their distribution across passerine hosts. Haplotypes that occur more than once are included in the table.

Haplotypes	COCCOC feces	ACRPAL feces	ACRSCH feces	ACRSCI feces	PARMAJ feces	CYACAE feces	ACRARU feces	FRICOE feces	ACRSCI blood	ACRSCH blood	ACRPAL blood	ACRARU blood	HIPICT blood	FRICOE blood	COCCOC blood	Total Seque nces
Hap_1	-	-	-	-	-	-	-	-	1	-	2	-	-	-	-	3
Hap_2	-	2	-	1	-	-	-	-	5	4	5	-	2	-	-	19
Hap_3	-	1	-	1	-	-	-	-	3	5	4	2	5	-	-	21
Hap_4	-	1	-	1	-	-	-	-	5	-	9	-	-	-	-	16
Hap_5	-	-	1	-	-	-	-	-	-	1	-	-	-	-	-	2
Hap_15	8	-	-	-	-	-	-	-	-	-	-	-	-	-	-	8
Hap_16	2	-	-	-	-	-	-	-	-	-	-	-	-	-	-	2
Hap_17	2	-	-	-	-	-	-	-	-	-	-	-	-	-	1	3
Hap_22	-	-	-	-	4	-	-	-	-	-	-	-	-	-	-	4
Hap_25	-	-	-	-	-	2	-	-	-	-	-	-	-	-	-	2

Table S3. *Lankesterella* lineages from the haplotype network (**Figure S1**) in our study that match with lineages from Chagas et al. (2021) in passerines of the Acrocephalidae species.

**Bird family and species**	**Lineages (Chagas et al. 2021)**	**Haplotype**
A. palustris	LanAcro12	Hap_10
	LanAcro3	Hap_10
	LanAcro11	Hap_1
A. schoenobaenus	LanAcro10	Hap_5
	LanAcro1	Hap_5
	LanAcro6	Hap_5
	LanAcro8	Hap_5
	LanAcro2	Hap_3
	LanAcro7	Hap_9
A. scirpaceus	LanAcro4	Hap_6
	LanAcro5	Hap_1
A. arundinaceus	LanAcro9	Hap_29

Table S4. Prevalences of *Lankesterella* and *Isospora* in infrequent hosts. Prevalences are based on microscopical records from host caught at different localities in Czechia.

**Avian host**	**Examined**	**Positive**	**Prevalence**	**Parasite genus**
Coccothraustes coccothraustes	451	1	0.2	*Isospora*
Emberiza citrinella	137	1	0.7	*Isospora*
Fringilla coelebs	167	1	0.6	*Isospora*
Garrulus glandarius	3	1	-	*Lankesterella*
Hirundo rustica	43	1	2.3	*Lankesterella*
Hippolais icterina	32*	9*	28*	*Isospora*
Phylloscopus collybita	46	1	2.2	*Lankesterella*
Sturnus vulgaris	70	1	1.4	*Isospora*
Sylvia atricapilla	78	1	1.3	*Isospora*

*PCR; none of the PCR positive samples had a microscopically positive slide

Table S5. Model passerine hosts and other infrequent hosts of avian *Lankesterella* and *Isospora* parasites used in the study and sample type.

Abbreviation	Species	Common name	Sample (*Lankesterella*)	Sample (*Isospora*)
ACRARU	*Acrocephalus arundinaceus*	great reed warbler	blood	blood and feces
ACRPAL	*Acrocephalus palustris*	marsh warbler	blood	blood and feces
ACRSCH	*Acrocephalus schoenobaenus*	sedge warbler	blood	blood and feces
ACRSCI	*Acrocephalus scirpaceus*	reed warbler	blood	blood and feces
CARTRI	*Carduelis tristis*	American goldfinch	_	blood
COCCOC	*Coccothraustes coccothraustes*	hawfinch	_	blood and feces
CURCOM	*Curruca communis*	common whitethroat	blood	_
CYACAE	*Cyanistes caeruleus*	blue tit	blood	feces
DELURB	*Delichon urbicum*	common house martin	blood	_
EMBCIT	*Emberiza citrinella*	yellowhammer	_	brain/liver tissue
ERIRUB	*Erithacus rubecula*	European robin	_	brain/liver tissue, blood
FRICOE	*Fringilla coelebs*	Eurasian chaffinch	_	blood and feces
GARGAL	*Garrulus glandarius*	Eurasian jay	blood	_
GYMTIB	*Gymnorhina tibicen*	Australian magpie	_	feces
HIPICT	*Hippolais icterina*	icterine warbler	blood	blood
HIRRUS	*Hirundo rustica*	barn swallow	blood	_
LAMSUR	*Lamprotornis superbus*	superb starling	_	feces
LANCOL	*Lanius collurio*	red-backed shrike	blood	_
MOTALB	*Motacilla alba*	white wagtail	blood	_
NEOTEM	*Neochmia temporalis*	red-browed finch	_	feces
PARMAJ	*Parus major*	great tit	blood	feces
PASCYA	*Passerina cyanea*	indigo bunting	_	feces
PASDOM	*Passer domesticus*	house sparrow	_	brain/liver tissue
PASMON	*Passer montanus*	Eurasian tree sparrow	_	brain/liver tissue
PHOOCH	*Phoenicurus ochruros*	black redstart	_	liver tissue
PHYCOL	*Phylloscopus collybita*	common chiffchaff	blood	_
PHYTRO	*Phylloscopus trochilus*	willow warbler	_	feces
PLENIV	*Plectrophenax nivalis*	snow bunting	blood	_
POEMON	*Poecile montanus*	willow tit	blood	_
POEPAL	*Poecile palustris*	marsh tit	blood	feces
SERCAN	*Serinus canaria*	Atlantic canary	_	feces
STRVER	*Strepera versicolor*	Grey currawong	_	feces
STUVUL	*Sturnus vulgaris*	common starling	blood	feces
SYLATR	*Sylvia atricapilla*	Eurasian blackcap	_	liver tissue
TROTRO	*Troglodytes troglodytes*	Eurasian wren	_	feces
TURFAL	*Turdus flacklandii*	Austral thrush	_	blood
TURMER	*Turdus merula*	blackbird	blood	_
ZOSLAT	*Zosterops lateralis*	silvereye	_	feces
